# Inattention in Obsessive-Compulsive Disorder Depends on Anxiety Load

**DOI:** 10.1192/j.eurpsy.2026.10639

**Published:** 2026-07-31

**Authors:** N. Abuasad, A. Adarsh, K. Nguyen, M. Grados, O.-C. G. A. S. (OCGAS)

**Affiliations:** 1 The Hashemite University, Zarqa, Jordan; 2 Shaheed Nirmal Mahto Medical College and Hospital, Jharkhand, India; 3 Johns Hopkins University; 4 Johns Hopkins University School of Medicine, Baltimore, United States

## Abstract

**Introduction:**

Obsessive-compulsive disorder (OCD) is diagnosed by clinically impairing obsessions and/or compulsions. A proportion of OCD is comorbid with attention-deficit/hyperactivity disorder (ADHD), especially with the inattentive subtype (ADHD-IA). Inattention in OCD has been explained due to a cognitive overload with anxiety and obsessions, impeding effortful concentration. This study explores whether a diagnosis of OCD with ADHD-IA is accompanied by excess anxiety, compared to OCD without ADHD-IA.

**Objectives:**

1) To test if anxiety disorders are in excess in OCD + ADHD-IA compared to OCD only; 2) to identify specific anxiety disorders that are in excess in OCD + ADHD-IA.

**Methods:**

989 probands from the OCGAS sample with a primary OCD diagnosis and ages 7-75 years comprised the sample. The Kiddie Schedules for Affective Disorders and Schizophrenia (K-SADS) was used to ascertain diagnoses of OCD and anxiety disorder in youth, and the Structured Clinical Interview for DSM Disorders (SCID) ascertained diagnoses in adults. Chi-square tests tested frequency data, and logistic regressions controlled for confounders.

**Results:**

Of 989 participants, there were 806 OCD only (81 %) and 183 OCD + ADHD-IA (19 %). OCD + ADHD-IA was more male (55% vs. 42%, p < 0.001), and younger (24.6 ± 13.3 vs. 30.3 ± 14.6 years, p= 0.002). OCD + ADHD-IA had a higher proportion of any anxiety disorder (73% vs. 52%; ▫▫2=27.2; p < 0.001), with the highest differences present for generalized anxiety disorder (GAD) (38% vs. 27%; ▫▫2 = 8.21; p = 0.004), social anxiety disorder (SocAnx) (30% vs. 20%; ▫▫2 = 7.24; p = 0.007), separation anxiety disorder (SAD) (31% vs. 16%; ▫▫2 = 22.54; p < 0.001) and specific phobia (SPho) (22% vs. 13%; ▫▫2 = 8.54; p = 0.003), but not for agoraphobia/panic disorder.

On stratification, adults with OCD + ADHD-IA had significantly more ‘any anxiety disorder’, SocAnx, and SAD, while youth had significantly more ‘any anxiety disorder’, GAD and SPho. Males with OCD + ADHD-IA had more ‘any anxiety disorder’, GAD and SAD, while females with OCD + ADHD-IA had more ‘any anxiety disorder’, SocAnx, SAD. Results did not change when logistic regressions controlled for age and sex of participants. Across groups, SAD had the strongest association with OCD + ADHD-IA.

**Conclusions:**

In this sample of OCD families recruited for genetic studies, probands with OCD with ADHD-IA had an excess of anxiety disorders with specific disorders linked to age and sex of participants. Whether the ADHD-IA phenotype is an alternate expression of anxiety in this OCD sample remains to be explored. In practice, both anxiety and ADHD-IA conflate the symptom of lack of concentration or inattention and may reflect the same mechanism of lack of effortful concentration.

**Disclosure of Interest:**

None Declared

